# Comprehensive profiling of Epstein-Barr virus-encoded miRNA species associated with specific latency types in tumor cells

**DOI:** 10.1186/1743-422X-10-314

**Published:** 2013-10-26

**Authors:** Hong-Jie Yang, Tie-Jun Huang, Chang-Fu Yang, Li-Xia Peng, Ran-Yi Liu, Guang-Da Yang, Qiao-Qiao Chu, Jia-Ling Huang, Na Liu, Hong-Bing Huang, Zhen-Yu Zhu, Chao-Nan Qian, Bi-Jun Huang

**Affiliations:** 1 State Key Laboratory of Oncology in South China; Collaborative Innovation Center for Cancer Medicine, Sun Yat-sen University Cancer Center, Guangzhou 510060, China; 2 Department of Nuclear Medicine, the second People’s Hospital of Shenzhen, Shenzhen 518038, China; 3 Department of Cancer Chemotherapy, the People’s Hospital of Gaozhou, Guangzhou, Guangdong province 525200, China; 4 Department of Biochemistry & Molecular Biology, Zhongshan School of Medicine, Sun Yat-sen University, Guangzhou Guangdong province, P.R. China; 5 Department of Medicine, Division of Infectious Diseases, University of Pennsylvania School of Medicine, Philadelphia, PA 19104-6073, USA; 6 School of Pharmaceutical Sciences, Sun Yat-sen University, Guangzhou People’s Republic of China

**Keywords:** Epstein-Barr virus, Viral microRNA, Latency types, Nasopharyngeal carcinoma, Burkitt’s lymphoma

## Abstract

**Background:**

Epstein-Barr virus (EBV) is an etiological cause of many human lymphocytic and epithelial malignancies. EBV expresses different genes that are associated with three latency types. To date, as many as 44 EBV-encoded miRNA species have been found, but their comprehensive profiles in the three types of latent infection that are associated with various types of tumors are not well documented.

**Methods:**

In the present study, we utilized poly (A)-tailed quantitative real-time RT-PCR in combination with microarray analysis to measure the relative abundances of viral miRNA species in a subset of representative lymphoid and epithelial tumor cells with various EBV latency types.

**Results:**

Our findings showed that the miR-BHRF1 and miR-BART families were expressed differentially in a tissue- and latency type-dependent manner. Specifically, in nasopharyngeal carcinoma (NPC) tissues and the EBV-positive cell line C666-1, the miR-BART family accounted for more than 10% of all detected miRNAs, suggesting that these miRNAs have important roles in maintaining latent EBV infections and in driving NPC tumorigenesis. In addition, EBV miRNA-based clustering analysis clearly distinguished between the three distinct EBV latency types, and our results suggested that a switch from type I to type III latency might occur in the Daudi BL cell line.

**Conclusions:**

Our data provide a comprehensive profiling of the EBV miRNA transcriptome that is associated with specific tumor cells in the three types of latent EBV infection states. EBV miRNA species represent a cluster of non-encoding latency biomarkers that are differentially expressed in tumor cells and may help to distinguish between the different latency types.

## Background

Increasing evidence suggests that EBV is an oncogenic human virus that causes many malignancies [1-3]. The EBV-encoded LMP1 gene is considered a viral oncogene that leads to the development of several tumor types [4,5]. Viral microRNA species have been found in EBV-infected cells [6] and recent studies indicate that 44 EBV miRNA species are involved in the establishment of viral latency and tumorigenesis [7-12]. However, the comparable profiling of EBV miRNA transcriptomes in tumor cells in the three types of latency states has not been performed. In this study, we investigated the comprehensive expression patterns of EBV miRNA species in cells with different types of latency and emphasized the viral miRNA patterns in NPC tissues and cells in the EBV latency II state.

MiRNAs play physiological and pathophysiologic roles in cell proliferation, differentiation and apoptosis by regulating protein expression in a post-transcriptional manner. Furthermore, miRNA regulation is an innate defense mechanism against viruses in host cells. Over the course of evolution, a subset of viruses, including the herpesviruses, has obtained the ability to hijack this antiviral mechanism of host cells to help maintain a persistent infection [13-16]. Approximately 20% of human tumors are associated with latent viral infections, and this latency is controlled by various viral genetic and epigenetic regulatory mechanisms [15,16]. Virus-encoded miRNAs play significant roles in maintaining persistent EBV infections by down-regulating the expression of viral immediate-early genes [7,17-19] and promoting the tumorigenesis of host cells. In addition, these viral miRNAs help the virus evade host immune defenses [9,11,20,21] by regulating host gene expression at the post-transcriptional level.

The classification of EBV latency is an academic and clinical issue because latent infections of different types are associated with different tumors [15]. The traditional classification system is based on only a few molecular markers of EBV latency, including EBERs, EBNA1-6, LMP1, LMP2 and BARTs [2,22-25], and typing of EBV latency types has limitations. Since EBV was first found to encode viral miRNAs in 2004 [6], 44 mature, EBV-encoded miRNA species have been registered in the Sanger miRBase library (Release 16.0). EBV miRNA species are produced from a large intron in BARTs (BamHI A rightward transcripts) prior to splicing [26,27]. Therefore, these novel, abundant markers of viral latency may help in obtaining further insight into EBV and may be useful as novel, biomarkers for identifying latency types [12,28].

MiRNAs are a class of difficult-to-detect RNA strands that are 19–23 nt in length. These RNAs can currently be analyzed by high-throughput microarray analysis [29,30], SOLEXA sequencing [31], Northern blotting [32] and quantitative real-time RT-PCR [33]. Given the higher sensitivity and specificity of qPCR [34], in this study, we mainly used a poly(A)-tailed, qRT-PCR technique in combination with a microarray assay to analyze the expression patterns of the EBV miRNA transcriptomes that are associated with specific latency types in various tumor cells. Additionally, we primarily evaluated a type I to III transition based on clustering analysis of the EBV miRNA species.

## Results

### Quantitative analysis of viral-coding transcripts in EBV-infected cell lines and NPC tissues

**Figure 1:**
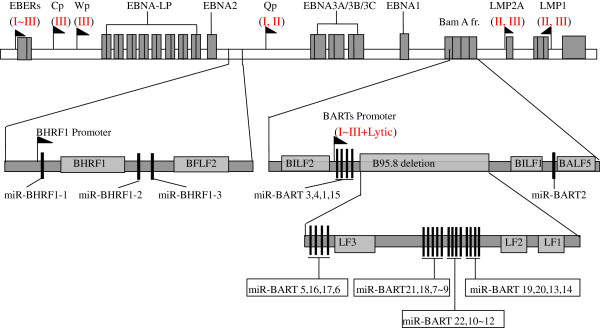
**Genomic locations of all viral miRNA species.** The top panel shows the position of latent genes and promoters (pennants). The middle panel shows the position of the EBV miRNAs, including the miR-BHRF1 and miR-BART families. The promoters of the BHRF1 and BART families are marked with pennants, and the EBV miRNA species whose genes are located within the deletion region in the prototype, B95-8-derived strain are shown in the lower panel. The locations of EBV miRNA genes are listed in genomic order from miRBase.

The previous identification of the EBV latency types was mainly based on viral EBERs, nuclear antigens, LMP1, LMP2 and the Bam H1 rightward transcripts [2,22-25]. EBNA1 is expressed under the alternative promoters Wp, Cp and Qp in a latency type-specific manner [22,24,35], and LMP1 exhibits differential abundance when compared to the various latency types [1], suggesting that the activities of the three latency promoters that control EBNA1 expression and LMP1 abundance can characterize the three latency types. The essential latency characteristics described above are partially illustrated in Figure 1. In the present study, we measured the activities of the Cp/Qp/Wp promoters and the expression level of LMP1 to characterize the different types of latent EBV infections.

**Figure 2:**
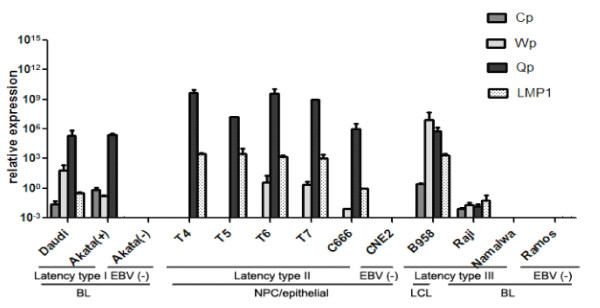
**Characteristic expression patterns of EBV latency biomarkers for viral-coding genes.** The levels of EBNA1 transcripts that were derived from promoters Q (Qp), C (Cp), and W (Wp) and the expression of LMP1 (latent membrane protein 1) were detected in cells having the three different EBV latency types by quantitative RT-PCR. Daudi and Akata(+) cells are EBV-positive BL (Burkitt’s lymphoma) cells with type I latency. T4, T5, T6 and T7 are pathologically confirmed, clinical NPC (nasopharyngeal carcinoma) specimens with EBV type II latency, and the C666-1 cell line is an epithelial NPC cell line that consistently harbors EBV. The Raji and Namalwa cell lines are EBV latency III BL cell lines, and the B95-8 cell line is a macaque lymphoblastoid cell line (LCL) harboring a prototype EBV strain. Akata(−), CNE2 and Ramos cells were used as EBV-negative controls, each qPCR reaction was run in triplicate, and the relative expression levels were calculated using the comparative method (2-ΔΔCt) with normalization to GAPDH.

We examined the expression levels of the traditional latency biomarkers by quantitative real-time RT-PCR in a subset of typical tumor cell lines and NPC tissues with different EBV latency types. During latency I (Daudi and Akata(+) cells) [36,37] and latency II (C666-1 and T4-T7 cells) [38], Qp exhibited a much higher activity than in latency III (B95-8, Raji and Namalwa cells). When compared with high-stringent latency I (Akata(+) cells), latency II (C666-1 and T4-7 cells) and latency III (B95-8 and Raji cells) were associated with slightly higher expression levels of LMP1. During low-stringent latency I (Daudi), the expression of the latency II/III biomarker LMP1 appeared to be elevated. Interestingly, viral latency biomarkers were nearly undetected by both methods in Namalwa cells, implying that the majority of the EBV episomes might be lost during culture (Figure 2).

### The comprehensive expression patterns of EBV miRNA species in three types of latency states

EBV miRNA species are expressed as two independent transcripts, BHRF1 and BART. The miR-BHRF1 family is located within the introns of BHRF1 (Bam HI fragment H rightward open reading frame 1) [10,39,40], whereas the miR-BART family is located in the intronic regions of the BART and form two separate clusters (cluster I and cluster II) [26,27]. The prototypic EBV strain that was derived from the B95-8 cell line has a 12-kb deletion that spans the two BART clusters from miR-BART-5 to 14 [41,42]. A detailed overview of the genomic locations of the EBV miRNA genes is presented in Figure 1.

**Figure 3:**
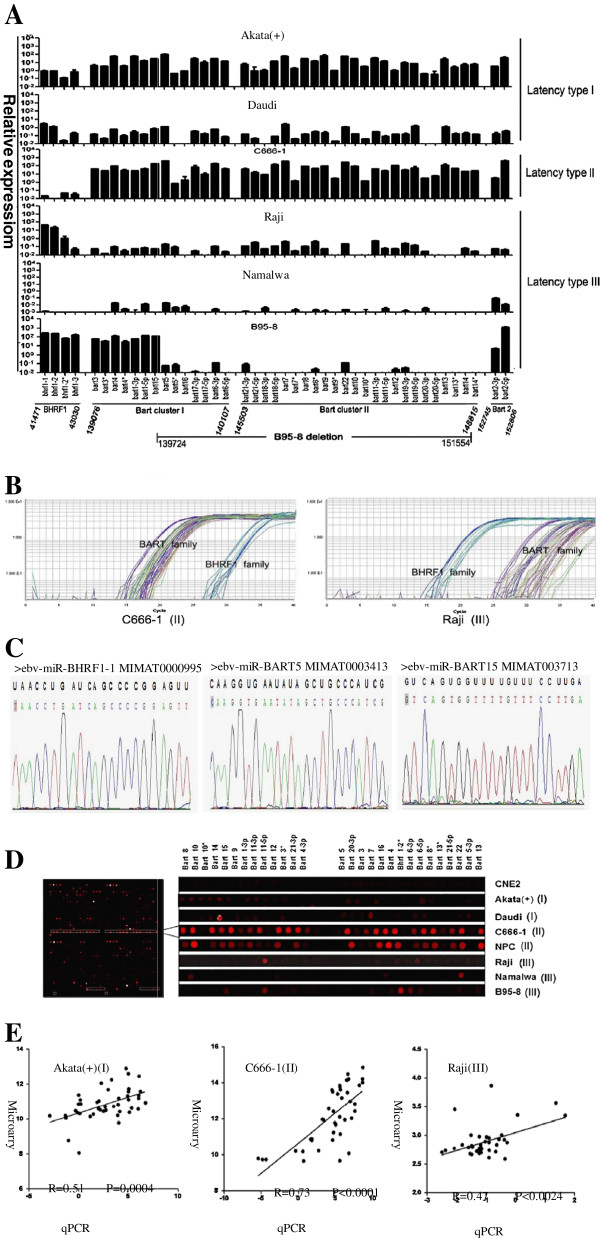
**Comprehensive and quantitative profiling of the EBV miRNA transcriptome in cells with the three latency types. (A)** The comparable results from poly(A)-tailed, real-time RT-PCR analysis of the EBV-encoded miRNA transcriptome in the cell lines with latency I, II and III. The respective positions of the four clusters of EBV miRNAs (miR-BHRF1, miR-BART clusters I/II, miR-BART2 and the B95-8 deletion region) are marked below, and the numbers under the lines indicate the EBV genomic locations (nt). **(B)** Differential amplification curves of EBV miRNA species between epithelial and lymphoid tumor cells. The amplification curves of EBV miRNAs in C666-1 and Raji cells are separated distinctly into two parts: in epithelial C666-1 cells, the EBV BART family generally had lower Ct values than the BHRF1 family (left); and the opposite results were observed for the lymphoid Raji cells (right). **(C)** Verification of qPCR product specificity via DNA sequencing based on AT cloning. Representative DNA sequence results are shown for three, randomly selected qPCR products, the BHRF1 family member BHRF1-1 and two BART family members, Bart5 and Bart15. **(D)** An overview image from the microarray analysis of human miRNAs and EBV miRNAs in C666-1 cells (left). The right panel displays a subset of the hybridization signals for EBV miRNAs in the indicated region of the chips for tumor cell lines with various EBV latency types and including the EBV-negative cell line CNE2. **(E)** Correlation between the microarray read counts and the expression levels that were determined by real-time PCR of EBV miRNAs that were detected in cells with various latency types. The microarray and real-time PCR results were log2-transformed and analyzed using Person’s correlation analysis. Pearson’s correlation coefficients and p-values are shown in the inserts.

In our investigation, we mainly used real-time RT-PCR to gain insight into the comparable profiles of the EBV miRNA transcriptomes of the three latency types. To confirm the specificity of the PCR products and verify the reliability of this poly(A)-tailed, real-time qPCR method, we randomly sequenced three qPCR products, submitted them to AT cloning procedures, and the results of DNA sequencing are shown in Figure 3C. Following qPCR analysis, we further evaluated the expression levels of EBV miRNAs using a microarray assay, and the scanning images are shown in Figure 3D. In addition, correlation analysis showed statistically significant correlations between the two methods for the latency I (Akata(+) cells; Pearson’s correlation coefficient = 0.51, p = 0.0004), latency II (C666-1 cells; Pearson’s correlation coefficient = 0.73, p < 0.0001) and latency III profiles (Raji cells; Pearson’s correlation coefficient = 0.47, p = 0.0024) (Figure 3E).

The quantitative analysis of the EBV miRNAs in each latency type yielded a comprehensive expression pattern of the EBV miRNA species (Figure 3A). Generally, the BHRF1 family exhibited a nearly 100-fold higher abundance in latency III (B95-8 and Raji cells) than in latency I (Akata(+) and Daudi cells), whereas the expression of the BHRF1 family was nearly absent in latency II (C666-1 cells), suggesting that the expression of the BHRF1 family is latency-III dependent. In addition, the BART family was differentially expressed in cells with the three latency types (Akata(+), Daudi, C666-1, Raji and B95-8 cells), with the exception of Namalwa cells, suggesting that the BART family plays differential roles in tumors with the three types of latency. Notably, the average expression level of the BART family in the epithelial tumor cell line C666-1, which is a latency II cell line, was the highest of all latency types, indicating that the BART family may contribute more to NPC tumorigenesis. In contrast, the expression level of the BHRF1 family was significantly higher than that of the BART family in the lymphoid tumor cell line Raji, which is a latency III cell line, suggesting that the BART and BHRF1 families are somewhat tissue-specific (Figure 3B). Interestingly, the BART and BHRF1 families were nearly undetectable in Namalwa cells, suggesting that EBV episomes might have been lost by these host cells in vitro.

Although a large difference between the expression levels of the BHRF1 and BART families was found in the same cell line, the difference between BART clusters I and II was relatively small in Akata(+), Daudi, C666-1 and Raji cells. This result suggests that BART clusters I and II are spliced from a common transcript, as was reported previously. Notably, 31 members of the EBV miRNA species that were barely detectable by qPCR are located in the 12-kb deletion in the prototypic B95-8-derived EBV strain that spans nts 139724–151554 of the EBV genome (GenBank accession number AJ507799.2). A detailed schematic of the deletion region in the B95-8-derived strain is shown in Figure 1. A subset of B95-8 cells can produce infectious EBV virions that are released into the medium during culture, which is a sign that the EBV life cycle has switched from latency to lysis. This switch might be related to the miRNAs that are encoded in the deleted region. That is, the miRNAs in the 12-kb deletion region could be involved in maintaining EBV latency. As for Akata(+) cells, which are high-stringent latency I cells, the abundance of the BHRF1 family was 10- to 100-fold lower than that of the BART family and was similar to its abundance in the high-stringent latency II cell line C666-1 (Figure 3A). Recent studies on the EBV methylome have found that hypermethylation of the promoter of the BHRF1 gene leads to its low expression in Akata cells, which is consistent with our present results [43].

### EBV miRNA transcriptome in primary NPC tissues and C666-1 cells with type II latency

**Figure 4:**
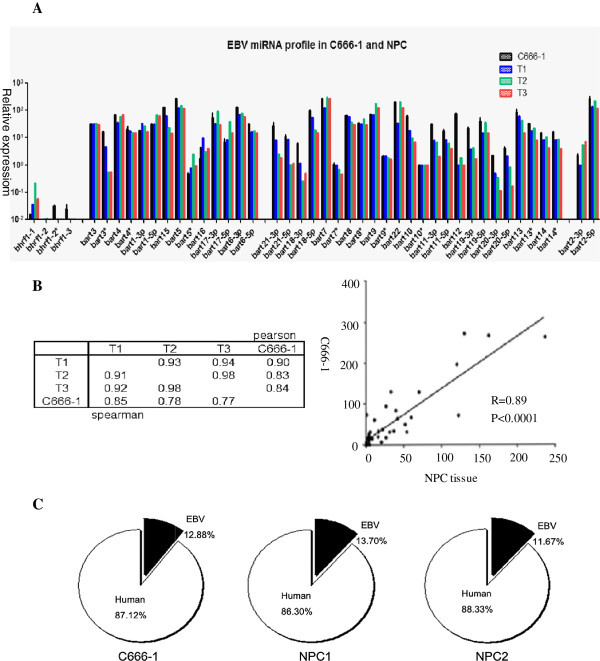
**The pattern of the EBV miRNA transcriptome in NPC cells and tissues in the latency II state. (A)** RT-PCR analysis of the EBV microRNA transcriptome in the persistently EBV-positive cell line C666-1 and three NPC tissue samples. U6 was used as an internal control. The whole EBV miRNAome is grouped into four clusters based on genomic location in sequential order. **(B)** Spearman and Pearson correlations between the C666-1 cells and the three NPC tissue samples (left). The expression levels of the EBV miRNAs were quantified by real-time RT-PCR. The data shown in the lower left portion are Pearson’s correlation coefficients, and the values shown in the upper right portion are Spearman’s correlation coefficients. To calculate the correlations, the real-time PCR results for the NPC tissues and C666-1 cells were log2-transformed and analyzed using Person’s correlation analysis. Pearson’s correlation coefficients and p-values are shown. The average expression level of each EBV miRNA species in the three NPC tissues is plotted on the x-axis (right). **(C)** Proportions of the respective human and EBV miRNA species that were detectable in C666-1 cells (left) and two NPC samples (middle and right) based on the microarray analysis. The EBV-encoded miRNAs accounted for more than 10% of the entire human miRNA library in the NPC samples in vivo and in vitro.

NPC tissues and the NPC cell line C666-1, which was the only cell line that persistently carried EBV, exhibited high-stringent, type II EBV latency [2,25,38,44]. In this study, we explored the EBV miRNA expression status of pathologically confirmed, NPC biopsy specimens, and our results indicated that the BHRF1 family was expressed at a low level, whereas the BART family showed much higher abundance in NPC samples and C666-1 cells (Figure 4A). The Pearson and Spearman correlation analyses of BART miRNA expression of these NPC samples and the C666-1 cell line showed great similarity, suggesting that the mechanism by which the EBV BART family regulates latency is the same in different NPC tumors (Figure 4B). The average expression level of the BART family in the NPC tissue samples also showed a strong correlation with its expression level in the NPC cell line C666-1 (R = 0.89, *p* < 0.0001) (Figure 4B). These results show that the EBV miRNA expression level in C666-1 cells is consistent with its pattern in NPC tissues, indicating that the C666-1 cell line is an ideal model for investigating the relationship between EBV and NPC. In addition, results of previous investigations on the pattern of EBV miRNA expression in NPC tissues using a stem-loop, RT primer-based qPCR assay are generally in agreement with our present results. We also evaluated the ratio of viral miRNAs to human miRNAs using the microarray data, and we found that the ratio was greater than 10% (Figure 4C). This result was also consistent with those of previous studies [31,45,46] and further indicates that EBV miRNAs might play important roles in NPC tumorigenesis.

### Evaluation of a possible transition from type I to III latency in Daudi cells that is dependent on viral miRNAs

**Figure 5:**
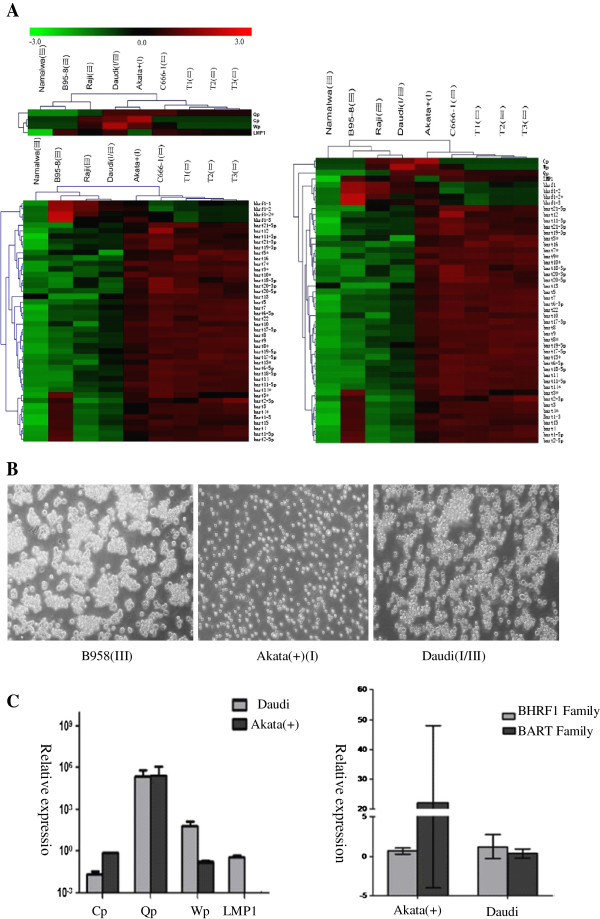
**Clustering analysis of the EBV miRNA transcriptome helps to identify a latency switch. (A)** Clustering analysis of the three latency types based on traditional EBV latency biomarkers (the EBNA1 promoters Cp, Wp, and Qp and LMP1) indicated that Daudi cells belonged to the EBV latency I group (upper left). However, unsupervised clustering analysis based on all of the EBV miRNA species alone (lower left) and in combination with classical latency biomarkers (right) suggested that Daudi cells might have undergone a transition from latency I to III. The three heat maps above were constructed based on normalized qPCR data. **(B)** Phenotypic observation of the Daudi cells in culture. Bright-field photomicrograph of three BL cell lines: B95-8 (left), Akata(+) (middle) and Daudi (right). The Daudi cells grew as macroscopic clumps, as was observed for the LCL B95-8 cells (the latency III control), whereas Akata(+) cells (latency I control) grew as a single-cell suspension. **(C)** Genotypic evaluation of the latency transition in Daudi cells. In contrast to Akata(+) cells, which exhibit latency I, Daudi cells had slightly higher levels of EBNA1 Wp activity and LMP1 expression (left). In addition, the average expression level of the BHRF1 family was slightly higher than that of the BART family in Daudi cells, which was in contrast to their patterns in Akata(+) cells (right).

The viral miRNAs that are differentially expressed in different types of EBV-infected cells are expected to help distinguish between the different latency types. In this study, we performed unsupervised, clustering analysis based on the qPCR dataset to examine the two types of indicators that are used to type latency. Interestingly, the human Burkitt’s lymphoma cell line Daudi [36,37], which was classified into the latency I group based on latency markers (Cp, Wp, Qp and LMP1), was classified into the latency III group based on the EBV miRNA latency markers (Figure 5A). Additionally, clustering analyses based on non-encoding miRNA species and their encoding latent genes categorized Daudi cells into the latency III group (Figure 5A). To evaluate the accuracy of the latter two clustering results, we performed phenotypic and genotypic investigations. Based on phenotypic characteristics (shown in Figure 5B), we observed that, in culture, Daudi cells grew as macroscopic clumps that were similar to those of the typical latency III cell line B95-8, whereas the high-stringent, latency I Akata(+) cells grew as a single-cell suspension, which is a typical characteristic of BL cells in latency I. The same phenomenon regarding the Daudi cell line was also observed in a recent study [12], suggesting that the EBV latency type might have switched from type I to III during the course of long-term culturing of Daudi cells. Additionally, we examined a latency type-specific change at the viral gene level when comparing high-stringent latency I Akata(+) cells, which have no LMP1 expression and lower Wp activity, to Daudi cells, which began to express the type III-associated marker LMP1 and had elevated Wp activity despite the persistence of higher Qp activity (Figure 5C). In addition to the changes in the encoding latency biomarkers, changes in the expression levels of viral non-coding biomarkers, including the BHRF1 and BART families, occurred in Daudi cells. In the representative type I cell line Akata(+), the average expression level of the BART family was much higher than that of the BHRF1 family, whereas the BHRF1 family had a slightly higher abundance than the BART family in Daudi cells, indicating that a transition from type I to type III latency had possibly occurred in the tested Daudi cells (Figure 5C). It seems that the limited number of traditional viral latency biomarkers is inadequate to clearly distinguish between the differential EBV latency types, whereas a cluster of differentially expressed miRNA species maybe reinforce these demands.

### Characterization of latent EBV coding and noncoding transcripts in cells and NPC tissues

The different EBV latency types are closely related to various malignancies, and, consequently, the correct identification of latency types is critical for understanding the virus’s contribution to carcinogenesis. EBV-encoded miRNAs were first found in 2004 [6], and, currently, as many as 44 EBV miRNA species have been found and registered in the Sanger miRBase. In this study, which is based on our comprehensive and comparable investigation of most EBV latency biomarkers, including coding and noncoding transcripts of the three latency types, we provide additional information on the in-depth characterization of EBV latent infections, as summarized in Table 1. In light of our analysis of viral latency biomarkers, these viral, non-encoding miRNA species should be considered when classifying the latency types of EBV-infected tumor cells.

**Table 1:** Characterization of EBV coding and noncoding transcripts in cells and NPC tissues tested

**Latency type**	**Cell line/tissue**	**Coding mRNA**				**Non-coding miRNAs**	
		**LMP1**	**EBNA1**	**Promoter**	**Activity**	**BHRF1 family**	**BART family**
			**Cp**	**Wp**	**Qp**		
EBV-positive							
I	Akata(+)(BL)	-	Low	Low	High	+	++
I/III	Daudi(BL)	+	Low	Medium	High	++	+
II	C666-1	+	None	Low	High	+	+++
	NPC tissues	+					
III	Raji(BL)	+	None	Medium	Low	+	+/-
	Namalwa(BL)	+/-	None	Low	None	+/-	-
	B95-8(LCL)	++	None	High	Medium	++	+
EBV-negative							
	CNE2(NPC)	-	-	-	-	-	-
	Ramos(BL)	-	-	-	-	-	-
	Akata(-)(BL)	-	-	-	-	-	-

Note: The abundance of all EBV latent coding and noncoding transcripts in the table above was determined through two different quantitative real time RT-PCR procedures respectively (see Materials and methods).

The abbreviation in the bracket indicates the tumor from which the cell lines were established with the exception to LCL. NPC: Nasopharyngeal carcinoma; BL: Burkitt’s lymphoma; LCL: Lymphoblastoid cell line.

-, negative; +/-,+,++ ,+++ indicate the level of EBV latent transcripts in an increasing order.

## Discussion

In light of the roles of human miRNAs under physiological and pathological conditions, especially in the process of carcinogenesis [47], the discovery of EBV-encoded miRNAs is an important event and a milestone in molecular virology that provided us with new insight into the relationship between a virus and its host cell. Earlier investigations showed that, in addition to EBV, other viruses such as HSV1/2 [19,48], HCMV [18], SV40 [21], KSHV [32] and even HIV [49] encode their own miRNAs, suggesting that, during long-term evolution, some viruses have hijacked this innate antivirus mechanism to serve themselves or to manipulate human cellular miRNAs to target viral genes [50]. Theoretically, the ability of a subset of viruses to encode their own miRNAs is an adaptive response to environmental stress because of the low immunogenic risk to the virus that is posed by those noncoding miRNAs [16,51].

Epstein-Barr virus infects over 90 percent of adults worldwide, and EBV infections are strongly associated with multiple human diseases, especially some cancers. In tumor cells, EBV genomes exist as episomes in a state of latency that does not produce lytic viral particles that can be detected by the host immune system. The EBV genome encodes approximately 90 genes but only expresses a few genes, such as EBERs, EBNA1-6, LMP1, LMP2 and BARTs [25], in latent stages, and these genes serve as markers for classical latency typing [23-25]. Based on the differential expression patterns of these viral genes, latent infections are divided into three types. In addition, tumors have different latency types, and the latency type can change during in vivo and in vitro culture [12,52]. Since the identification of EBV-encoded miRNAs in 2004 [6], researchers have increasingly focused their attention on investigating the relationships between viral miRNAs and the establishment of EBV latency and host carcinogenesis.

In this study, we analyzed the comprehensive expression patterns of EBV miRNA species using quantitative real-time PCR and microarray assays in representative tumor cell lines with the three latency types. The cell lines included the type I latency cell lines Akata(+) and Daudi, the type II latency cell line C666-1 and the type III latency cell lines Namalwa, Raji and B95-8, and we analyzed several NPC tissues. In addition, we explored the profile of the EBV miRNA transcriptome in-depth. Generally, the expression of the BHRF1 miRNA family is dependent on the type of viral latency and the cell’s histological origin, whereas the BART miRNA family is expressed differentially in cells during all forms of latency. Moreover, qPCR analysis indicated a difference of nearly 100-fold between all tested cell lines, and the expression of individual BART miRNA species within one cell line could differ by 50-fold or more, although those miRNAs were transcribed together as a single, primary transcript [27]. In contrast, the BART family was expressed constantly at a high level without a significant difference between its expression in EBV-positive NPC tissues and the C666-1 cell line (latency II), suggesting that the BART family is involved in maintaining EBV latency and promoting NPC tumorigenesis. In fact, recent research has demonstrated that the BART family plays important roles in the establishment of EBV latency and NPC initiation and development. Noticeably, ebv-miR-bart6-5p can target the human dicer gene to control the latency switch from type I/II to type III [52]. Briefly, our results and emerging evidence indicate that EBV-encoded miRNA species have a functional role in target gene regulation during carcinogenesis in host cells.

NPC, which is prevalent in southern China, is a typical model of type II EBV latency, in which only three protein-coding genes are expressed: EBNA1, LMP1 and LMP2A. Our results indicate that the noncoding BART family was expressed highly and accounted for more than 10% of all known human miRNAs, suggesting that, like the viral oncogene LMP1, these abundant noncoding miRNA species are likely involved in NPC pathogenesis. As in other viruses, the use of miRNAs is one of the strategies used by the virus to evade host-immune defenses because noncoding miRNAs, which have no immunogenicity, cannot trigger the body’s immune response. Therefore, the production of miRNAs is a relatively low-risk and efficient regulatory mechanism for maintaining a persistent infection and stability of its genome [53]. In addition, we found that the EBV miRNA transcriptome was somewhat stable in the NPC tissues and the type II latency cell line C666-1, which further suggested that the EBV BART family plays key roles in virus latency and host tumorigenesis [54]. The results also imply that the NPC cell line C666-1 is an appropriate cellular model to examine the EBV infection status in vivo and is an ideal cell line for exploring the relationship between EBV and NPC [38].

It is generally agreed that Burkitt’s lymphoma exhibits EBV type I latency in vivo and that NPC exhibits type II latency [44]. Latency I and II are low-risk types because fewer immunogenic viral proteins are expressed during those times than during high-risk latency III [52]. EBV-positive cells cultured in vitro are not under surveillance by the immune system of the host; therefore, these cells experience little or no immune pressure, which may result in the loss of the virus or a change in its latency type. This could explain why EBV is missing from nearly all NPC cell lines, with the exception of the C666-1 cell line. Similarly, lymphoma cells in long-term culture occasionally lose EBV episomes, as was observed in the Namalwa BL cell line in this study, where the BHRF1 and BART families were expressed at very low levels (Figure 3A and D). Moreover, EBV can spontaneously switch latency types in some lymphoma cells in vitro, and the most common switch is a type I-to-type III transition [12]. However, certain subtle latency transitions are difficult to detect and identify due to the absence of more specific and sensitive latency biomarkers. Because EBV miRNA species are a cluster of novel latency markers consisting of 44 members, including the BHRF1 family and the BART family, that are differentially expressed in host cells, we profiled the patterns of the EBV miRNA transcriptome in tumor cells with the three different latency types, and we expected that clustering analysis would distinguish between the three latency types. We primarily observed a latency switch from type I to type III in the Daudi BL cell line, which was previously regarded to be a cell line in the latency I state, during the clustering analysis, and we further investigated this switch by phenotypic and genotypic analyses. It seems that these numerous, noncoding viral miRNA species might be informative and valuable for determining latency type.

## Conclusions

Viral noncoding miRNAs seem to be preferential regulatory tools that are extensively utilized by EBV due to their low immunogenicity. Thus, EBV is a good model for studying the role of viral miRNA regulation in the maintenance of latency. Our results suggest that these differentially expressed viral miRNAs are molecular biomarkers of latent infection and may be useful in identifying latency types. In particular, the BART family contributes greatly to epithelial tumors and to some lymphomas, whereas the BHRF1 family is primarily responsible for a subset of lymphocyte tumors. Our data provide fine profiles of the EBV miRNA transcriptomes that are associated with the three EBV latency types and suggest that different viral miRNA species are involved in various host-cell carcinogenesis processes.

## Materials and methods

### Cell culture and tissues

The cell lines and tissues used in this study are listed in Table 2. The Daudi, Raji and B95-8 cell lines were obtained from the American Type Culture Collection (http://www.atcc.org). The Namalwa, Akata, Ramos and CNE2 cell lines were kindly provided by Prof. Musheng Zeng (Cancer Center, Sun Yat-Sen University), and the C666-1 cell line was a gift from The Chinese University of Hong Kong (CUHK, Hong Kong SAR, China) [38]. All of the cells were maintained in RPMI media with 10% fetal bovine serum (Gibco, USA) and 1% penicillin-streptomycin and were incubated in 5% CO2 at 37°C in a humidified atmosphere. The primary NPC tissues used in this research were biopsies obtained from the Cancer Center at Sun Yat-Sen University (Guangzhou, China) prior to any treatments and were pathologically confirmed as nondifferentiated non-keratinizing carcinomas (UNKC, WHO type III). This project was performed in agreement with the clinical ethics guidelines, and all clinical samples were collected and analyzed with prior written, informed consent from the patients. This project was approved by the Research Ethics Committee of the Sun Yat-Sen University Cancer Center.

**Table 2:** Histopathological and EBV infectious characterization of all cell lines and cancer tissues used in this study

**Cell line/Tissue**	**Pathology/Cell type**	**EBV status**	**Latency type**	**Reference**
CNE2	NPC/epithelial	-	-	
Ramos	BL/B lymphocytic	-	-	[55]
Akata(-)	BL/B lymphocytic	-	-	[56]
Daudi	BL/B lymphocytic	+	I	[36]
Akata(+)	BL/B lymphocytic	+	I	[56]
C666-1	NPC/epithelial	+	II	[38]
Raji	BL/B lymphocytic	+	III	[57]
Namalwa	BL/B lymphocytic	+	III	[58]
B95-8	LCL/B lymphoblastoid	+	III	[59]
T1	NPC(UNKC)/epithelial	+	II	
T2	NPC(UNKC)/epithelial	+	II	
T3	NPC(UNKC)/epithelial	+	II	
T4	NPC(UNKC)/epithelial	+	II	
T5	NPC(UNKC)/epithelial	+	II	
T6	NPC(UNKC)/epithelial	+	II	
T7	NPC(UNKC)/epithelial	+	II	

NPC: Nasopharyngeal carcinoma.

UNKC: Undifferentiated non-keratinizing carcinoma.

BL: Burkitt lymphoma.

LCL: Lymphoblastoid cell line.

T1-7: pathologically confirmed NPC biopsy specimens.

### Small RNA-enriched total RNA isolation

Total RNA containing low-molecular-weight RNAs was prepared using the TRIzol method with minor modifications. Briefly, to extract more small RNAs, following the first precipitation, the resulting upper aqueous phase was mixed with 0.5 ml pre-cooled, isopropyl alcohol per 1 ml of TRIzol Reagent, and this mixture was then placed at −20°C for 45 min and then spun at 12,000 × g at 4°C for 45 min. The pellets were washed in 75% ethanol and then allowed to air dry for 5 min. The RNA pellets were resuspended in nuclease-free water, quantified by UV spectrophotometry (Nanodrop, Thermo, USA), aliquoted and stored at −80°C.

### Quantitative RT-PCR analysis of viral-coding gene expression

The three EBNA1 promoter activity patterns (known as Wp, Cp and Qp) and LMP1 expression were analyzed by quantitative RT-PCR assays using an ABI7900HT Fast Real-Time PCR System (Applied Biosystems, USA). The housekeeping gene GAPDH was used as an internal control to verify the RNA quality and loading accuracy. Reverse transcription of 2 μg of total RNA was performed using M-MLV reverse transcriptase (Promega, USA) according to the manufacturer’s guidelines. The EBV-negative tumor cell lines Akata(−), CNE2 and Ramos were used as negative controls for type I, II and III latency, respectively. In the qPCR assay, each reaction was run in triplicate tubes in the presence of SYBR green I, and the raw Ct values were normalized to that of the housekeeping gene GAPDH. The PCR primer sequences for all analyzed genes are listed in Additional file 1: Table S1. The DNA oligonucleotides that were used as PCR primers and the chip probes in the study were synthesized and HPLC-purified by Invitrogen Inc. (Guangzhou, China).

### Poly (A)-tailed, quantitative real-time RT-PCR analysis of the EBV miRNA species

The All-in-One miRNA qRT-PCR Detection Kit (GeneCopoeia, USA) was used to determine the miRNA levels in the samples. Briefly, poly(A) polymerase was used to add poly(A) tails to the 3′ ends of the miRNAs, and M-MLV RTase and a unique oligo-dT adaptor primer were used to transcribe the poly(A)-tailed miRNAs to generate a cDNA library. The All-in-One qPCR Mix containing SYBR green I along with the miRNA-specific primers and the general primer were used to produce the respective cDNAs for all EBV-encoded miRNA species using an ABI7900HT Fast Real-Time PCR System (Applied Biosystems, USA). Thermal cycling was performed as follows: initial denaturation at 95°C for 10 min; 40 cycles of denaturation at 95°C for 10 s, annealing at 60°C for 20 s and extension at 72°C for 10 s; and a final extension at 72°C for 10 min (GeneCopoeia, USA). The specificity of the qPCR products was evaluated and then verified by melting curve analysis, gel electrophoresis and AT cloning-based DNA sequencing (pMD18-T, TAKARA, Japan). The genomic locations of the EBV miRNA genes are illustrated in Figure 1, and all of the miRNA-specific forward primers that were used are listed in Additional file 1: Table S2. The EBV-negative NPC cell line CNE2 was used as a negative control, and the housekeeping gene U6 was used as an internal control for normalization. Each qPCR reaction was run in triplicate, and the relative expression levels were calculated using the comparative method (2^-ΔΔCt^) [34].

### Microarray evaluation of human and viral miRNA expression

The oligonucleotide microarray for the analysis of microRNAs was fabricated in-house, as described previously with minor modifications [60]. Briefly, pre-cleaned, Gold Seal slides (Thermo Fisher Scientific Inc.) were washed with detergents and baked at 140°C for 4 hours, and then the probes (40 nM final concentration) were mixed with printing buffer at a 1:4 ratio and were printed onto the slides at a constant temperature and humidity using a SmartArrayer 136 printer (CapitalBio Inc., Beijing, China). The microRNA probe set included human and EBV miRNAs that were registered in Sanger miRBase Release 12.0 (http://www.mirbase.org), and several positive and negative controls were designed and labeled, in part, according to other published documents. The EBV miRNA probe sequences are listed in Additional file 1: Table S2. The probe labeling and hybridization were performed, in part, as described previously with minor modifications [30,61]. Briefly, 2.5 μg of total RNA was labeled with 2 nmol of pCp-DY647 or pCp-DY547 (Dharmacon, USA) using 15 units of T4 RNA ligase (GE Healthcare, USA) in a total reaction volume of 20 μL at 16°C overnight. The resulting 2.5 μg of labeled, small RNA-enriched total RNA was purified using Bio-Spin 6 desalting columns (BIO-RAD, USA) and then condensed in vacuum. These miRNAs were hybridized to pretreated chips in 1× hybridization solution (5× Denhardt’s solution, 0.5% SDS, 5× SSC) in a hybridization chamber at 46°C for 24 hours, which was followed by slide washing and scanning with a LuxScan™ 10 K Microarray Scanner (CapitalBio Inc., China). Subsequently, the scanned images were analyzed using GenPix Pro 6.0 (Axon Instruments, U.S.A.), all data were MIAME compliant, and the raw data have been deposited into a MIAME-compliant database (GSE33225).

### Clustering analysis

Given the higher sensitivity, specificity and reliability of qPCR analysis compared to microarray evaluation, we performed unsupervised clustering analysis using the normalized qPCR data [28,62]. The tumor cell lines and tissues with different EBV latency types were clustered using TMEV (TIGR multiarray experiment viewer, version 4.2.01, http://www.tm4.org/) based on the EBV miRNA expression levels that were quantified by poly(A)-tailed, quantitative real-time RT-PCR, and the data were log2 transformed. The hierarchical clustering of the EBV miRNA species and the classical typing markers, including Cp, Wp and Qp activity and LMP1 expression, was performed using average linkage clustering, and the Euclidian distance matrix was calculated.

## Competing interests

The authors declare that they have no competing interests.

## Authors’ contributions

HBJ and QCN contributed to the conception of the idea, design, data collection, drafting and writing of the manuscript. YHJ, HTJ and YCF contributed to conception of the idea, design and writing of the manuscript. YCF and YGD contributed to data collection and culture of the cells. CQQ and HJL contributed to the laboratory work and drafting of the manuscript. LRY and PLX contributed to data analysis and writing of the manuscript. HHB and ZZY contributed to conception of the idea, data collection and writing of the manuscript. LN contributed to conception and writing of the manuscript. All read and approved the manuscript.

## Supplementary Material

Additional file 1: Table S1 The PCR primers sequences for viral latent genes. **Table S2.** The PCR primer and microarray probe sequences for EBV miRNA species. Click here for file
