# Supplementary material for: Comprehensive profiling of Epstein-Barr virus-encoded miRNA species associated with specific latency types in tumor cells
Source: Virol J. 2013 Oct 26;10:314. doi: 10.1186/1743-422X-10-314 (PMC4231337; doi:10.1186/1743-422X-10-314)
Supplement: Additional file 1: Table S1 — The PCR primers sequences for viral latent genes. Table S2. The PCR primer and microarray probe sequences for EBV miRNA species. [file 1743-422X-10-314-S1.pdf]

Supplementary Table 1 The PCR primers sequences for viral latent genes

Primer Name	Sequence
Cp forword primer	CATCTAAACCGACTGAAGAA
Cp reverse primer	CCCTGAAGGTGAACCGCTTA
Qp forword primer	TAGCGTGCGCTACCGG
Qp reverse primer	CATTTCCAGGTCCTGTACCT
Wp forword primer	GTCCACACAAATCCTAG
Wp reverse primer	CCCTGAAGGTGAACCGCTTA
GAPDH forword primer	CTCCTCCTGTTCGACAGTCAGC
GAPDH reverse primer	CCCAATACGACCAAATCCGTT

Note: The PCR primers refer to J Biol Chem. 2010 Oct 22;285(43):33358-70.

Supplementary Table 2 The PCR primer and microarray probe sequences for EBV miRNA species

ebv-miR	Oligo	Sequence
BHRF1-1	Location	41474-41495
	Mature Sequence	UAACCUGAUCAGCCCCGGAGUU
	Primer Sequence	TAACCTGATCAGCCCCGGAGTTAA
	Probe Sequence	GAACTCCGGGGCTGATCAGGTTA
BHRF1-2*	Location	42853-42874
	Mature Sequence	AAAUUCUGUUGCAGCAGAUAGC
	Primer Sequence	AAATTCTGTTGCAGCAGATAGCAA
	Probe Sequence	GGCTATCTGCTGCAACAGAATTT
BHRF 1-2	Location	42888-42909
	Mature Sequence	UAUCUUUUGCGGCAGAAAUUGA
	Primer Sequence	TATCTTTTGCGGCAGAAATTGAAA
	Probe Sequence	GTCAATTTCTGCCGCAAAAGATA
BHRF 1-3	Location	42968-42989
	Mature Sequence	UAACGGGAAGUGUGUAAGCACA
	Primer Sequence	TAACGGGAAGTGTGTAAGCACAAA
	Probe Sequence	GTGTGCTTACACACTTCCCGTTA
BART 3*	Location	139087-139107
	Mature Sequence	ACCUAGUGUUAGUGUUGUGCU
	Primer Sequence	ACCTAGTGTTAGTGTTGTGCTAA
	Probe Sequence	GAGCACAACACTAACACTAGGT
BART 3	Location	139125-139145
	Mature Sequence	CGCACCACUAGUCACCAGGUGU
	Primer Sequence	CGCACCACTAGTCACCAGGTGTAA
	Probe Sequence	GACACCTGGTGACTAGTGGTGCG

BART 4	Location	139228-139249
	Mature Sequence	GACCUGAUGCUGCUGGUGUGCU
	Primer Sequence	GACCTGATGCTGCTGGTGTGCTAA
	Probe Sequence	GAGCACACCAGCAGCATCAGGTC
BART 4*	Location	139266-139288
	Mature Sequence	CACAUCACGUAGGCACCAGGUGU
	Primer Sequence	CACATCACGTAGGCACCAGGTGTAA
	Probe Sequence	GACACCTGGTGCCTACGTGATGTG
BART 1-5p	Location	139351-139374
	Mature Sequence	UCUUAGUGGAAGUGACGUGCUGUG
	Primer Sequence	TCTTAGTGGAAGTGACGTGCTGTGAA
	Probe Sequence	GCCTGGACCTTGACTATGAAACA
BART 1-3p	Location	139387-139408
	Mature Sequence	UAGCACCGCUAUCCACUAUGUC
	Primer Sequence	TAGCACCGCTATCCACTATGTCAA
	Probe Sequence	GGACATAGTGGATAGCGGTGCTA
BART 15	Location	139553-139574
	Mature Sequence	GUCAGUGGUUUUGUUUCCUUGA
	Primer Sequence	GTCAGTGGTTTTGTTTCCTTGAA
	Probe Sequence	GTCAAGGAAACAAAACCACTGAC
BART 5	Location	139675-139698
	Mature Sequence	CAAGGUGAAUAUAGCUGCCCAUCG
	Primer Sequence	CAAGGTGAATATAGCTGCCCATCGAA
	Probe Sequence	GCGATGGGCAGCTATATTCACCTTG
BART 5*	Location	139717-139736
	Mature Sequence	GUGGGCCGCUGUUCACCU
	Primer Sequence	GTGGGCCGCTGTTCACCTAA
	Probe Sequence	GAGGTGAACAGCGGCCCAC
BART 16	Location	139795-139817
	Mature Sequence	UUAGAUAGAGUGGGUGUGUGCUCU
	Primer Sequence	TTAGATAGAGTGGGTGTGTGCTCTAA
	Probe Sequence	GAGAGCACACACCCACTCTATCTAA
BART 17-5p	Location	139915-139935
	Mature Sequence	UAAGAGGACGCAGGCAUACAAG
	Primer Sequence	TAAGAGGACGCAGGCATACAAGAA
	Probe Sequence	GCTTGTATGCCTGCGTCCTCTTA
BART 17-3p	Location	139953-139975
	Mature Sequence	UGUAUGCCUGGUGUCCCCUUAGU
	Primer Sequence	TGTATGCCTGGTGTCCCCTTAGTAA
	Probe Sequence	GACTAAGGGGACACCAGGCATACA
BART 6-5p	Location	140033-140054
	Mature Sequence	UAAGGUUGGUCCAAUCCAUAGG
	Primer Sequence	TAAGGTTGGTCCAATCCATAGGAA
	Probe Sequence	GCCTATGGATTGGACCAACCTTA

BART 6-3p	Location	140072-140093
	Mature Sequence	CGGGGAUCGGACUAGCCUUAGA
	Primer Sequence	CGGGGATCGGACTAGCCTTAGAAA
	Probe Sequence	GTCTAAGGCTAGTCCGATCCCCG
BART 21-5p	Location	145514-145534
	Mature Sequence	UCACUAGUGAAGGCAACUAAC
	Primer Sequence	TCACTAGTGAAGGCAACTAACAA
	Probe Sequence	GGTTAGTTGCCTTCACTAGTGA
BART 21-3p	Location	145548-145569
	Mature Sequence	CUAGUUGUGCCCACUGGUGUUU
	Primer Sequence	CTAGTTGTGCCCACTGGTGTTTAAA
	Probe Sequence	GAAACACCAGTGGGCACAACTAG
BART 18-5p	Location	145962-145983
	Mature Sequence	UCAAGUUCGCACUUCCUAUACA
	Primer Sequence	TCAAGTTCGCACTTCCTATACAAA
	Probe Sequence	GTGTATAGGAAGTGCGAACTTGA
BART 18-3p	Location	145998-146019
	Mature Sequence	UAUCGGAAGUUUGGGCUUCGUC
	Primer Sequence	TATCGGAAGTTTGGGCTTCGTCAA
	Probe Sequence	GGACGAAGCCCAAACTTCCGATA
BART 7*	Location	146439-146460
	Mature Sequence	CCUGGACCUUGACUAUGAAACA
	Primer Sequence	CCTGGACCTTGACTATGAAACAAA
	Probe Sequence	GTGTTTCATAGTCAAGGTCCAGG
BART 7	Location	146475-146496
	Mature Sequence	CAUCAUAGUCCAGUGUCCAGGG
	Primer Sequence	TCATAGTCCAGTGTCCAGGGAA
	Probe Sequence	GCCCTGGACACTGGACTATGATG
BART 8	Location	146772-146793
	Mature Sequence	UACGGUUUCCUAGAUUGUACAG
	Primer Sequence	TACGGTTTCCTAGATTGTACAGAA
	Probe Sequence	GCTGTACAATCTAGGAAACCGTA
BART 8*	Location	146807-146829
	Mature Sequence	GUCACAAUCUAUGGGGUCGUAGA
	Primer Sequence	GTCACAATCTATGGGGTCGTAGAAA
	Probe Sequence	GTCTACGACCCCATAGATTGTGAC
BART 9*	Location	146959-146980
	Mature Sequence	UACUGGACCCUGAAUUGGAAAC
	Primer Sequence	TACTGGACCCTGAATTGGAAACAA
	Probe Sequence	GGTTTCCAATTCAGGGTCCAGTA
BART 9	Location	146997-147019
	Mature Sequence	UAACACUUCAUGGGUCCCGUAGU
	Primer Sequence	TAACACTTCATGGGTCCCGTAGTAA
	Probe Sequence	GACTACGGGACCCATGAAGTGTTA

BART 22	Location	147203-147225
	Mature Sequence	UUACAAAGUCAUGGUCUAGUAGU
	Primer Sequence	CTTACAAAGTCATGGTCTAGTAGTAAAAA
	Probe Sequence	ACTACTAGACCATGACTTTGTAA
BART 10*	Location	147321-147342
	Mature Sequence	GCCACCUCUUUGGUUCUGUACA
	Primer Sequence	GCCACCTCTTTGGTTCTGTACAAA
	Probe Sequence	GTGTACAGAACCAAAGAGGTGGC
BART 10	Location	147356-147378
	Mature Sequence	UACAUAACCAUGGAGUUGGCUGU
	Primer Sequence	TACATAACCATGGAGTTGGCTGTAA
	Probe Sequence	GACAGCCAACTCCATGGTTATGTA
BART 11-5p	Location	147537-147560
	Mature Sequence	UCAGACAGUUUGGUGCGCUAGUUG
	Primer Sequence	TCAGACAGTTTGGTGCGCTAGTTGAA
	Probe Sequence	GCAACTAGCGCACCAAACTGTCTGA
BART 11-3p	Location	147575-147595
	Mature Sequence	ACGCACACCAGGCUGACUGCC
	Primer Sequence	ACGCACACCAGGCTGACTGCCAA
	Probe Sequence	GGGCAGTCAGCCTGGTGTGCGT
BART 12	Location	147936-147957
	Mature Sequence	UCCUGUGGUGUUUGGUGUGGUU
	Primer Sequence	TCCTGTGGTGTTTGGTGTGGTTAA
	Probe Sequence	GAACCACACCAAACACCACAGGA
BART 19-5p	Location	148215-148237
	Mature Sequence	ACAUUCCCCGCAAACAUGACAUG
	Primer Sequence	TTCCCCGCAAACATGACATGAA
	Probe Sequence	GCATGTCATGTTTGCGGGGAATGT
BART 19-3p	Location	148254-148274
	Mature Sequence	UUUUGUUUGCUUGGGAAUGCU
	Primer Sequence	TTTTGTTTGCTTGGGAATGCTAA
	Probe Sequence	GAGCATTCCCAAGCAAACAAAA
BART 20-5p	Location	148339-148359
	Mature Sequence	UAGCAGGCAUGUCUUCAUUCC
	Primer Sequence	TAGCAGGCATGTCTTCATTCCAA
	Probe Sequence	GGGAATGAAGACATGCCTGCTA
BART 20-3p	Location	148374-148395
	Mature Sequence	CAUGAAGGCACAGCCUGUUACC
	Primer Sequence	CATGAAGGCACAGCCTGTTACCAA
	Probe Sequence	GGGTAACAGGCTGTGCCTTCATG
BART 13*	Location	148526-148547
	Mature Sequence	AACCGGCUCGUGGCUCGUACAG
	Primer Sequence	AACCGGCTCGTGGCTCGTACAGAA
	Probe Sequence	GCTGTACGAGCCACGAGCCGGTT

BART 13	Location	148563-148585
	Mature Sequence	UGUAACUUGCCAGGGACGGCUGA
	Primer Sequence	TGTAACTTGCCAGGGACGGCTGAAA
	Probe Sequence	GTCAGCCGTCCCTGGCAAGTTACA
BART 14*	Location	148744-148765
	Mature Sequence	UACCCUACGCUGCCGAUUUACA
	Primer Sequence	TACCCTACGCTGCCGATTTACAAA
	Probe Sequence	GTGTAAATCGGCAGCGTAGGGTA
BART 14	Location	148778-148799
	Mature Sequence	UAAAUGCUGCAGUAGUAGGGAU
	Primer Sequence	TAAATGCTGCAGTAGTAGGGATAA
	Probe Sequence	GATCCCTACTACTGCAGCATTTA
BART 2-5p	Location	152747-152768
	Mature Sequence	UAUUUUCUGCAUUCGCCCUUGC
	Primer Sequence	TATTTTCTGCATTCGCCCTTGCAA
	Probe Sequence	GGCAAGGGCGAATGCAGAAAATA
BART 2-3p	Location	152783-152806
	Mature Sequence	AAGGAGCGAUUUGGAGAAAAUAAA
	Primer Sequence	AAGGAGCGATTTGGAGAAAATAAAAA
	Probe Sequence	GTTTATTTTCTCCAAATCGCTCCTT

Note: The genomic locations of EBV miRNA species refer to the positions in the EBV genome (GenBank accession number AJ507799.2). Sequences of mature miRNA species are taken from Sanger miRNA Registry (http://microrna.sanger.ac.uk). Primer sequence indicates specific qRT-PCR primer for each miRNAs and Probe sequence means microarray probe oligo nucleotide sequence designed for each EBV miRNAs.
